# Ultrasound-Assisted Covalent Conjugation of Walnut Albumin with Bound Polyphenols: Structural Modulation and Functional Enhancement

**DOI:** 10.3390/foods15112033

**Published:** 2026-06-05

**Authors:** Ziyu Li, Lifei Wei, Qiulan Shi, Yanju Xiang, Yunfeng Pu, Donghong Liu

**Affiliations:** 1 College of Food Science and Engineering, Tarim University, Alar 843300, China; 12413063@zju.edu.cn (Z.L.); 2023255@xjit.edu.cn (L.W.); xiangyanju@163.com (Y.X.); 2 National-Local Joint Engineering Research Center of Intelligent Food Technology and Equipment, Zhejiang Key Laboratory of Agro-food Resources and High-Value Utilization, Fuli Institute of Food Science, College of Biosystems Engineering and Food Science, Zhejiang University, Hangzhou 310058, China; 3 Future Food Laboratory, Innovation Center of Yangtze River Delta, Zhejiang University, Jiaxing 314100, China; 4 Production & Construction Group Key Laboratory of Special Agricultural Products Further Processing in Southern Xinjiang, Alar 843300, China; 5 Aral Quality and Technical Supervision Comprehensive Testing and Inspection Institute, Alar 843300, China; 15003031275@163.com

**Keywords:** walnut albumin, ultrasound-assisted covalent conjugation, bound polyphenols, protein–polyphenol interactions, functional properties

## Abstract

Background: Walnut albumin (WA) possesses a balanced amino acid composition but exhibits poor solubility, limited emulsifying capacity, and low structural stability, restricting its practical applications in food systems. Methods: In this study, bound polyphenols (BPs) from jujube pomace were covalently conjugated with WA through alkaline and radical methods, with or without ultrasound assistance. Four WA–BPs conjugates were prepared, including alkaline-treated (AWA–BPs), ultrasound-assisted alkaline (UAWA–BPs), radical-treated (RWA–BPs), and ultrasound-assisted radical conjugates (URWA–BPs), to investigate the effects of different covalent assembly methods on the structural and functional properties of WA. Results: Covalent conjugation with BPs significantly altered the structural properties of WA, as evidenced by reductions in reactive groups, changes in surface hydrophobicity, fluorescence quenching, and shifts in FTIR spectra. URWA–BPs exhibited the highest grafting degree of 4.46 mg/g dry weight (DW) among the four treatment groups, indicating effective grafting and conformational rearrangement. Moreover, URWA–BPs demonstrated superior functional properties, including improved solubility, emulsifying activity, foaming properties, and antioxidant capacity. The DPPH, ABTS^+^, and FRAP values of URWA–BPs increased by approximately 10–17% compared with WA. In contrast, UAWA–BPs exhibited the lowest in vitro digestibility (54.90 ± 1.60%), indicating enhanced structural stability against gastrointestinal digestion. Molecular docking revealed binding free energies ranging from −5.3 to −7.6 kcal/mol, suggesting stable interactions between BPs and WA. Conclusions: The differences observed between UAWA–BPs and URWA–BPs suggest that, in addition to promoting covalent conjugation, ultrasound exerts distinct regulatory effects during alkaline and radical covalent assembly processes, resulting in different structural and functional properties. This study provides new perspectives for designing functional plant-based protein ingredients and valorizing food-processing by-products.

## 1. Introduction

Walnut meal is an important by-product of walnut oil production and is rich in high-quality plant proteins, mainly including glutelin, albumin, globulin, and prolamin [1,2]. Although water-soluble walnut albumin (WA) is not the predominant protein fraction in walnuts, it has attracted considerable attention due to its favorable solubility, balanced amino acid composition, and high digestibility [3]. However, its limited emulsifying properties and antioxidant activities still restrict its broader applications in food systems. Therefore, effective strategies for structural modification and functional enhancement of WA are highly desirable. Polyphenols and proteins are widely distributed in food matrices, and their interactions not only affect the stability and bioavailability of polyphenols but also modulate the physicochemical and functional properties of proteins [4]. Jujube pomace, a major by-product of the jujube juice industry, is rich in polyphenols, mainly including free polyphenols (FPs) and bound polyphenols (BPs) [5]. Compared with FPs, which are generally present as low-molecular-weight and readily soluble compounds, BPs are a class of phenolic compounds covalently bound to cell wall components or other food matrices through ester, glycosidic, and ether linkages [6], and often exhibit a higher degree of polymerization and greater molecular heterogeneity. BPs can account for more than 60% of total polyphenols in many plant by-products, and their abundant phenolic hydroxyl groups and distinct structural features may provide multiple reactive sites for protein interactions, making them promising natural modifiers for food proteins [7]. However, the influence of these structural characteristics on protein–polyphenol covalent conjugation, particularly under ultrasound treatment, remains insufficiently understood [8]. Conventional methods for polyphenol extraction mainly include organic solvent extraction and reflux extraction; however, these approaches generally suffer from long extraction times, high energy consumption, and excessive solvent usage. In recent years, various green and efficient extraction technologies have been developed to improve the extraction efficiency of polyphenols and reduce environmental impact, such as ultrasound-assisted extraction, microwave-assisted extraction, and natural deep eutectic solvent (NADES)-based systems [9,10]. Among these, food-grade NADES have attracted considerable attention due to their environmental friendliness, low toxicity, and high extraction efficiency [11].

Polyphenols can associate with proteins through both non-covalent and covalent interactions, thereby significantly influencing protein structure and functional properties [12,13]. Non-covalent interactions mainly involve hydrophobic interactions, hydrogen bonding, electrostatic interactions, and van der Waals forces, whereas covalent interactions are typically initiated by the oxidation of polyphenols into reactive quinone intermediates, which subsequently react with nucleophilic groups such as amino and sulfhydryl groups in proteins. This process introduces phenolic moieties into protein structures, leading to changes in protein conformation, surface properties, and stability, thereby further improving their functional performance [14,15]. In addition, variations in polyphenol molecular structure, degree of polymerization, and distribution of reactive sites may further influence protein–polyphenol interaction patterns and binding behaviors [16]. For instance, soy protein isolate–polyphenol conjugates formed under alkaline conditions exhibit improved antioxidant activity [17]. Similarly, covalent binding with phenolic compounds such as anthocyanins has been shown to improve protein flexibility and structural stability, outperforming non-covalent interactions [18,19].

In recent years, ultrasound has emerged as an effective physical means to regulate protein structure and reaction processes. Ultrasound treatment can influence molecular interactions and reaction environments, where acoustic cavitation plays a critical role in promoting protein unfolding and modulating covalent assembly processes [20]. Compared with conventional methods, ultrasound may affect not only the extent of covalent conjugation but also the spatial distribution and connectivity of crosslinking networks, leading to distinct structural and functional outcomes [21,22]. For example, Wei et al. [23] recently reported that ultrasound-assisted covalent conjugation between walnut protein isolates and EGCG significantly enhanced polyphenol grafting efficiency and induced pronounced structural–functional transformations. Consistently, Wu et al. [24] further demonstrated that walnut protein–polyphenol conjugates prepared using different strategies, including physical mixing, heat treatment, alkali treatment, and ultrasound-assisted free-radical grafting, exhibited distinct physicochemical, structural, and functional characteristics, emphasizing the critical role of conjugation methods in determining the properties of the resulting conjugates. However, the differential effects of ultrasound on distinct covalent assembly routes, particularly in multicomponent BP systems characterized by higher degrees of polymerization and diverse reactive sites, remain insufficiently understood.

This study aimed to investigate the effects of ultrasound-assisted alkaline and radical covalent conjugation of BPs with WA on the structural, functional, and digestive properties of the resulting complexes. BPs were extracted from jujube pomace via ultrasound-assisted extraction and characterized by HPLC, while WA was isolated from walnut meal and its amino acid composition was determined. WA–BP conjugates were prepared using alkaline and radical methods, with ultrasound assistance. Structural characteristics were analyzed using surface hydrophobicity, intrinsic fluorescence spectroscopy, SEM, particle size, and FTIR. Furthermore, the functional differences among conjugates prepared by different coupling strategies were evaluated in terms of antioxidant activity, solubility, emulsifying capacity, and in vitro digestibility. Molecular docking was also performed to elucidate the interaction mechanisms between representative phenolic compounds and WA at the molecular level.

## 2. Materials and Methods

### 2.1. Materials

Jujube fruit (*Ziziphus jujuba* Mill. cv. Junzao) was purchased from a local market in Alar, Xinjiang, China. Walnut meal was obtained from a walnut oil processing factory in Hotan, Xinjiang, China. Gallic acid, rutin, protocatechuic acid, catechin, p-hydroxybenzoic acid, epicatechin, p-coumaric acid, quercetin, ascorbic acid, polyphenol oxidase, L-lysine, and glycine were purchased from Shanghai Yuanye Bio-Technology Co., Ltd. (Shanghai, China). All other reagents used in this study were of analytical grade or higher.

### 2.2. Extraction and Characterization of BPs

**Figure 1:**
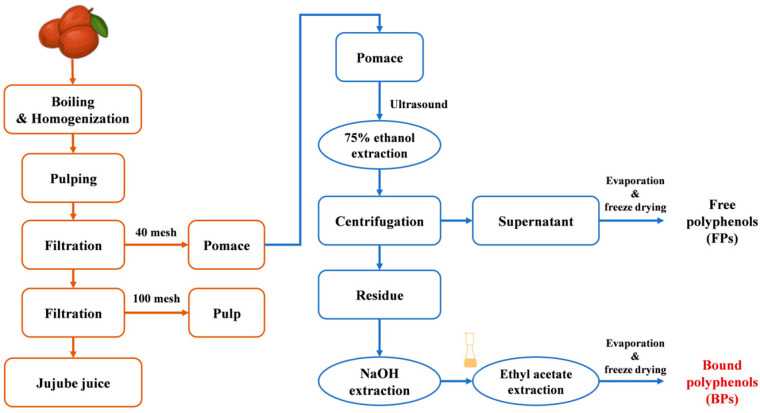
Processing flowchart for the extraction of BPs from jujube pomace.

The processing workflow for jujube pomace preparation and BP extraction is shown in Figure 1. Jujube fruits were boiled, homogenized, filtered, and dried at 60 °C to obtain jujube pomace. FPs were first removed using 75% (*v*/*v*) ethanol (S/L ratio of 1:20 g/mL) with ultrasonic assistance, following Pan et al. [25]. The remaining residue was subsequently subjected to ultrasound-assisted alkaline extraction based on Sun et al. [26]. After alkaline treatment, the extract was acidified to pH 2.0, followed by ethyl acetate extraction. The organic phase was then evaporated and freeze-dried to obtain BPs.

The phenolic composition of BPs was analyzed by HPLC according to Pu et al. [27] with minor modifications. Chromatographic separation was performed on a Spuirsil C18 column (250 × 4.6 mm, Dima Technology, Tianjin, China) using methanol (A) and 0.5% formic acid in water (B) as the mobile phases. Detection wavelengths were set at 280 and 360 nm. The detailed gradient program is provided in Table S1.

### 2.3. Preparation and Characterization of WA

#### 2.3.1. Preparation of WA

Walnut meal was ground, sieved, and mixed with deionized water at a S/L ratio of 1:15 (*w*/*v*). The mixture was stirred at room temperature for 2 h and centrifuged at 15,000× *g* for 25 min at 4 °C. The supernatant was collected as the WA fraction. The precipitate was re-extracted once under the same conditions, and the supernatants were combined and dialyzed against deionized water using a dialysis membrane (MWCO: 3.5 kDa, Beijing Solarbio Science & Technology Co., Ltd., Beijing, China) at 4 °C for 48 h, with the dialysis medium renewed every 8 h. The retentate was subsequently freeze-dried and stored at 4 °C.

#### 2.3.2. Amino Acid Analysis of WA

The amino acid composition of WA was analyzed using an automated amino acid analyzer (LA8080, Hitachi High-Technologies Corporation, Tokyo, Japan) according to the method reported by Dias and de Moura Bell [28]. WA powder (0.03 g) was hydrolyzed in 10 mL of 6 mol/L HCl at 110 °C for 22–24 h. The hydrolysate was concentrated under reduced pressure to remove acid, diluted to 5 mL with 0.01 mol/L HCl, filtered, and subjected to analysis.

### 2.4. Preparation of WA–BPs

Four types of WA–BPs were prepared using alkaline and radical modification methods, either in the absence or presence of ultrasound, including alkaline-treated WA–BPs (AWA–BPs), ultrasound-assisted alkaline WA–BPs (UAWA–BPs), radical-treated WA–BPs (RWA–BPs), and ultrasound-assisted radical WA–BPs (URWA–BPs). Ultrasonic treatment was conducted using a probe sonicator (SCIENTZ-IID, Ningbo Scientz Biotechnology Co., Ltd., Ningbo, China) at a fixed power of 200 W for 10 min at room temperature. All other conditions were kept identical to ensure comparability among treatments.

AWA–BPs: WA (1.0 g) was dissolved in deionized water, and the pH was adjusted to 9.0. The solution was stirred overnight at 4 °C, and an equal volume of BP solution was added. The mixture was reacted at room temperature for 24 h while maintaining pH 9.0. The product was dialyzed against deionized water using a 3.5 kDa molecular weight cutoff (MWCO) membrane at 4 °C for 48 h (dialysate renewed every 6 h) and freeze-dried to obtain AWA–BPs.

UAWA–BPs: The preparation procedure was similar to that of AWA–BPs, except that the mixture was subjected to ultrasonic treatment (200 W, 10 min) after the addition of BPs and before the 24 h reaction at room temperature.

RWA–BPs: WA (1.0 g) was dissolved in 99.0 mL of deionized water and stirred overnight at 4 °C. The BP solution was then added, followed by the sequential addition of 1.0 mL of 5.0 M H_2_O_2_ and 0.25 g of ascorbic acid. Subsequently, the reaction was continued at room temperature for 24 h. The product was dialyzed against deionized water using a 3.5 kDa MWCO membrane at 4 °C for 48 h (dialysate renewed every 6 h) and freeze-dried to obtain RWA–BPs.

URWA–BPs: The preparation procedure was similar to that of RWA–BPs, except that the resulting mixture was subjected to ultrasonic treatment (200 W, 10 min) after the addition of H_2_O_2_ and ascorbic acid, followed by reaction at room temperature for 24 h.

### 2.5. Reactive Groups and Grafting Degree Measurement of WA–BPs

#### 2.5.1. Free Amino Groups

The content of free amino groups (–NH_2_) before and after reaction was determined using the o-phthalaldehyde (OPA) method [29]. The sample solution (0.2 mg/mL, 0.2 mL) was mixed with OPA reagent (4 mL) and incubated at 35 °C for 2 min. The absorbance was measured at 340 nm using distilled water as the blank. L-lysine was used as the standard for calibration.

#### 2.5.2. Free Sulfhydryl Groups

The free sulfhydryl group content was determined using Ellman’s method based on Li et al. [30]. Samples (10 mg) were dissolved in Tris–glycine buffer (pH 8.0), followed by the addition of Ellman’s reagent. After incubation at room temperature for 1 h in the dark, the absorbance was measured at 412 nm. The sulfhydryl content was calculated using the corresponding equation:

(1) $$\mathrm{SH}(\mu \mathrm{mol}/g)\;=\;73.53\;\times \;\frac{A_{412}}{C}$$

where A_412_ is the absorbance at 412 nm and C is the sample concentration (mg/mL).

#### 2.5.3. Tryptophan Residues

Freeze-dried samples were dissolved in deionized water to obtain protein solutions (0.5 mg/mL). A 0.9 mL sample solution was mixed with 1 mL of nitric acid (16.0 M) and heated in a water bath at 50 °C for 15 min. After rapid cooling, 4 mL of NaOH (5.0 M) and 4 mL of ethanol were added sequentially and thoroughly mixed. The absorbance was then measured at 360 nm and 430 nm. The tryptophan (Trp) content was calculated using the following equation:

(2) $$C=\;0.5357\;\times \;A_{430}-0.35714\;\times {\;A}_{360}$$

where C is the Trp concentration (μg/mL), and A_430_ and A_360_ are the absorbance values at 430 nm and 360 nm, respectively.

### 2.6. Structural Properties of WA–BPs

#### 2.6.1. Surface Hydrophobicity Measurement

Surface hydrophobicity (H_0_) was determined based on the method of Lee et al. [31] with slight modifications, using 8-anilino-1-naphthalenesulfonic acid (ANS) as a fluorescent probe. Protein solutions at different concentrations (0.05–0.25 mg/mL) were prepared in 0.02 M PBS (pH 8.0). Each sample (2.0 mL) was mixed with ANS solution (10 μL, 8 mM) and incubated in the dark at room temperature for 15 min. Fluorescence intensity was measured at an excitation wavelength of 370 nm and an emission wavelength of 470 nm. The initial slope of fluorescence intensity versus protein concentration was used to calculate H_0_.

#### 2.6.2. Intrinsic Fluorescence Spectroscopy Measurement

Sample solutions (0.25 mg/mL) were prepared in 0.02 M PBS (pH 8.0). Intrinsic fluorescence spectra were recorded at room temperature using a fluorescence spectrophotometer (27368, Thermo Fisher Scientific, Waltham, MA, USA), with an excitation wavelength of 280 nm, an emission range of 300–450 nm, and slit widths set to 5 nm.

#### 2.6.3. SEM Measurement

Samples were sputter-coated with gold and observed using a desktop SEM (Phenom ProX, Thermo Fisher Scientific, Eindhoven, The Netherlands). Images were acquired at 15.0 kV with a magnification of 5000×.

#### 2.6.4. Particle Size, PDI and Zeta Potential Measurements

Particle size was measured using a laser particle size analyzer (Mastersizer 2000, Malvern Instruments, Malvern, UK), while PDI and zeta potential were determined using a Zetasizer Nano ZS (Malvern Instruments, UK), according to the method of Dai et al. [32].

#### 2.6.5. FTIR Measurement

FTIR spectra were collected following the method of Song et al. [33]. Spectra were recorded using an FTIR instrument (IRTracer-100, Shimadzu Corporation, Kyoto, Japan) over 400–4000 cm^−1^ with 32 scans.

### 2.7. Functional Properties of WA–BPs

#### 2.7.1. Solubility Measurement

Samples were dissolved in deionized water to obtain a concentration of 1.0 mg/mL and stirred at room temperature for 1 h. The solution was centrifuged at 5000 rpm for 20 min, and the protein content of the supernatant was determined using the Coomassie Brilliant Blue method. Solubility was expressed as the ratio of protein concentration in the supernatant to the total protein concentration.

(3) $$\mathrm{Solubility}\;(\%)=\frac{\mathrm{Protein}\;\mathrm{content}\;\mathrm{in}\;\mathrm{supernatant}}{\mathrm{Total}\;\mathrm{protein}\;\mathrm{content}\;\mathrm{of}\;\mathrm{sample}}\;\times \;100$$

#### 2.7.2. Emulsifying Properties Measurement

The emulsifying properties were determined according to the method of Lin et al. [34]. Samples were dissolved in 0.01 M PBS buffer (pH 7.0), and 15 mL of the solution was mixed with 5 mL of soybean oil and homogenized at 13,500 rpm for 2 min. Emulsion samples (100 μL) were taken at 0 and 10 min, diluted 100-fold with 0.1% (*w*/*v*) sodium dodecyl sulfate (SDS) solution, and the absorbance was measured at 500 nm. The emulsifying activity index (EAI) and emulsifying stability index (ESI) were calculated using the following equations:

(4) $$\mathrm{EAI}(m^{2}/g)\;=\;\frac{2\;\times \;2.303\;\times \;A_{0}\;\times \;\varepsilon }{C\;\times \;\phi \;\times \;10^{4}}$$

(5) $$\mathrm{ESI}(\min)\;=\frac{A_{0\;}\times \;10}{\left(A_{0}-A_{10}\right)}$$

where A_0_ and A_10_ are the absorbance values of the emulsion at 0 and 10 min, respectively; c is the initial protein concentration (g/mL); and ϕ is the oil volume fraction (%).

#### 2.7.3. Foaming Properties Measurement

Foaming properties were evaluated following the method reported by Yang et al. [35]. Foaming capacity (FC) and foam stability (FS) were calculated as follows:

(6) $$\mathrm{FC}(\%)\;=\;\frac{V_{0}}{50}\;\times \;100$$

(7) $$\mathrm{FS}(\%)=\frac{V_{1}}{V_{0}}\;\times \;100$$

where V_0_ is the foam volume at 0 min (mL), and V_1_ is the foam volume at 30 min (mL).

#### 2.7.4. Water-Holding and Oil-Holding Capacity Measurement

The water-holding capacity (WHC) and oil-holding capacity (OHC) were determined according to a modified method. A 1.0 g sample (W_0_) was placed in a centrifuge tube and weighed (W_1_). Then, 20 mL of ultrapure water or soybean oil was added, mixed for 30 s, and allowed to stand for 30 min. The mixture was centrifuged at 10,000 rpm for 10 min at 4 °C, and the supernatant was discarded. The tube with sediment was weighed again (W_2_). WHC and OHC were calculated as follows:

(8) $$\mathrm{WHC}\;\mathrm{or}\;\mathrm{OHC}\;(g/g)\;=\;\frac{W_{2\;}-\;W_{1}}{W_{0}}$$

where W_0_ is the sample weight (g), W_1_ is the weight of the sample and centrifuge tube (g), and W_2_ is the weight of the sediment and centrifuge tube after centrifugation (g).

#### 2.7.5. Antioxidant Activity Measurement

The DPPH radical scavenging activity was determined according to the method of Gu et al. [36]. Equal volumes (2 mL) of sample solution and DPPH solution were mixed and incubated in the dark at room temperature for 30 min. Subsequently, 200 μL of the reaction mixture was transferred into a 96-well microplate, and the absorbance (A_1_) was measured at 517 nm using a microplate spectrophotometer (Multiskan GO, Thermo Fisher Scientific, USA). Ethanol instead of DPPH solution was used as the sample blank (A_0_), while ethanol instead of sample solution was used as the control (A_2_). The DPPH radical scavenging activity was calculated as follows:

(9) $$\mathrm{DPPH}\;\mathrm{radical}\;\mathrm{scavenging}\;\mathrm{rate}(\%)\;=\;(1\;-\;\frac{A_{1}\;-\;A_{0}}{A_{2}})\;\times \;100$$

The ABTS^+^ radical scavenging activity was determined following the method of Zhao et al. [37]. Sample solution (1 mL) was mixed with 3 mL of ABTS^+^ working solution and incubated at room temperature for 1 h. Subsequently, 200 μL of the reaction mixture was transferred into a 96-well microplate, and the absorbance (A_1_) was measured at 734 nm using a microplate spectrophotometer (Multiskan GO, Thermo Fisher Scientific, USA). PBS (pH 7.0) instead of ABTS^+^ working solution was used as the sample blank (A_0_), while PBS (pH 7.0) instead of sample solution was used as the control (A_2_). The ABTS^+^ radical scavenging activity was calculated as follows:

(10) $${\mathrm{ABTS}}^{+}\mathrm{radical}\;\mathrm{scavenging}\;\mathrm{rate}(\%)\;=\;(1\;-\;\frac{A_{1}\;-\;A_{0}}{A_{2}})\;\times \;100$$

The ferric reducing antioxidant power (FRAP) was evaluated according to the method of Tang et al. [38]. The FRAP reagent was prepared by mixing 255 mmol/L NaCl solution (in 178 mmol/L acetic acid), 10 mmol/L tripyridyltriazine (TPTZ) solution (in 40 mmol/L HCl), and 34 mmol/L FeCl_3_ solution at a ratio of 10:1:1 (*v*/*v*/*v*). Sample solution (0.2 mL) was then added to 1.8 mL of FRAP reagent and incubated in the dark at room temperature for 20 min. The absorbance was measured at 593 nm.

### 2.8. In Vitro Protein Digestibility Measurement of WA–BPs

In vitro protein digestibility of WA–BPs was evaluated following the simulated gastrointestinal digestion model reported by Fan et al. [39], which includes sequential oral, gastric, and intestinal digestion processes and has been widely applied for evaluating digestion behavior of protein–polyphenol systems. Samples (0.5 mg/mL) were mixed with simulated salivary fluid (SSF, containing 75 U/mL α-amylase and 1.5 mmol/L CaCl_2_) at a 1:1 (*v*/*v*) ratio, adjusted to pH 7.0, and stirred at 37 °C and 150 rpm for 2 min. Subsequently, simulated gastric fluid (SGF, containing 40 mg NaCl and 64 mg pepsin, adjusted to pH 1.2 with 5 mol/L HCl) was added at a 1:1 (*v*/*v*) ratio, and the mixture was incubated at 37 °C for 2 h. The resulting gastric digesta were then combined with simulated intestinal fluid (SIF, containing 40 mg bile salts, 400 mg trypsin, 0.67 g CaCl_2_·2H_2_O, 4 g NaCl, 0.1 g KCl, 575 mg Na_2_HPO_4_·12H_2_O, and 0.1 g KH_2_PO_4_, adjusted to pH 7.4) at a 1:1 (*v*/*v*) ratio and incubated at 37 °C for additional 2 h. After digestion, enzyme activity was terminated by heating the samples at 100 °C for 5 min. The intestinal digesta were mixed with 10% (*w*/*v*) trichloroacetic acid (TCA) at a 1:1 ratio and kept at 4 °C for 24 h, followed by centrifugation at 10,000 rpm for 30 min. The resulting precipitate was dissolved in 1 mL of 1 mol/L NaOH, and protein contents before and after digestion were determined using the bicinchoninic acid (BCA) method to calculate protein digestibility.

### 2.9. Molecular Docking

Molecular structures of p-coumaric acid, catechin, epicatechin, and quercetin were constructed using ChemDraw 23.1.1 and exported in PDB format. WA belongs to the typical 2S seed storage albumin family, with the major walnut allergen Jug r 1 as its representative member. Therefore, the predicted three-dimensional structure of Jug r 1 (UniProt ID: P93198) from the AlphaFold database was selected as the receptor protein. The receptor and ligands were then pre-processed using AutoDockTools 1.5.7 by removing water molecules, adding polar hydrogens, and converting the structures into PDBQT format. Molecular docking was performed using AutoDock Vina 1.1.2. Blind docking was carried out using a large grid box covering the entire receptor surface, with the grid center set to x = −4.307, y = −5.644, and z = 4.238 Å, and the grid size set to 47.25 × 47.25 × 47.25 Å. The docking conformation with the lowest predicted binding energy was selected for subsequent interaction analysis. The docking results were visualized using PyMOL 3.1.6.1.

### 2.10. Statistical Analysis

All measurements were conducted in triplicate, and results were presented as mean ± standard deviation. Statistical analysis was performed using one-way ANOVA with SPSS 26.0, followed by Duncan’s multiple range test to assess significant differences (*p* < 0.05). Figures were generated using Origin 2022.

## 3. Results

### 3.1. Ultrasound-Assisted Alkaline Extraction of BPs

To optimize the ultrasound-assisted alkaline extraction of BPs from jujube pomace, the effects of NaOH concentration, solid-to-liquid ratio, temperature, and ultrasonic power on BP yield were systematically evaluated (Figure S1). The optimal extraction conditions were determined as follows: NaOH concentration of 1.2 mol/L, solid-to-liquid ratio of 1:20 (g/mL), extraction temperature of 80 °C, and ultrasonic power of 200 W. Under these conditions, the absorbance value obtained in the validation experiment was 0.443 ± 0.009, corresponding to an extraction yield of 0.81 ± 0.01 mg/g DW (Tables S2 and S3).

HPLC analysis of the BPs identified eight major phenolic constituents, including gallic acid, protocatechuic acid, catechin, p-hydroxybenzoic acid, epicatechin, p-coumaric acid, rutin, and quercetin (Figure S2; Table S4). Phenolic acids and flavan-3-ols were predominant, indicating a structurally diverse polyphenolic composition.

### 3.2. Amino Acid Composition of WA

WA was extracted from walnut meal (Table S5) and subsequently characterized to evaluate its structural basis for covalent conjugation with BPs. The amino acid composition analysis (Table S6) revealed 17 amino acids, including seven essential amino acids. Importantly, WA contains multiple nucleophilic residues, such as lysine, cysteine, and aromatic amino acids, which provide potential reactive sites for covalent conjugation with polyphenols. Tryptophan was not detected due to degradation during acid hydrolysis [40,41].

### 3.3. Reactive Groups and Grafting Degree of WA–BPs

**Figure 2:**
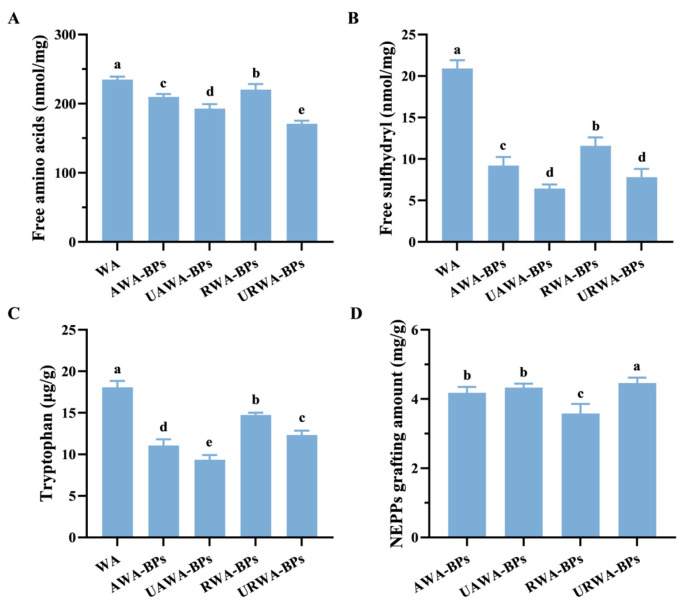
Free amino groups (–NH_2_) (**A**), free sulfhydryl groups (**B**), tryptophan (Trp) content (**C**), and BP grafting amount (**D**) of WA–BPs. Different letters indicate significant differences among samples (*p* < 0.05).

Polyphenols are readily oxidized into quinone intermediates, which subsequently react with nucleophilic groups in proteins to form covalent conjugates. Considering that ultrasound may enhance the reaction process through cavitation-induced structural unfolding and mass transfer, this study compared the effects of different preparation strategies and ultrasound treatments on WA–BPs by determining the contents of –NH_2_, Trp, and free sulfhydryl groups, together with the grafting amount of polyphenols using the Folin assay. As shown in Figure 2A–C, all conjugates exhibited significantly lower levels of –NH_2_, Trp, and free sulfhydryl groups compared with WA (*p* < 0.05). This result agrees with that of Li et al. [13], who found similar covalent interactions between coffee protein and 5-caffeoylquinic acid. Liu et al. [42] reported that whey protein–polyphenol conjugation reduced –NH_2_ and sulfhydryl levels and increased the molecular weight of the complexes. Among all samples, URWA–BPs exhibited the lowest contents of –NH_2_ and Trp, followed by UAWA–BPs, AWA–BPs and RWA–BPs. Ultrasound treatments further decreased reactive group contents compared with non-ultrasound samples, suggesting enhanced exposure and subsequent reaction of nucleophilic sites.

As shown in Figure 2D, the grafting amounts of BPs for AWA–BPs, UAWA–BPs, RWA–BPs, and URWA–BPs were 4.11 ± 0.15, 4.40 ± 0.12, 3.58 ± 0.28, and 4.46 ± 0.15 mg/g DW, respectively, with URWA–BPs exhibiting the highest grafting level. Wu et al. [43] reported that polyphenol–lactoferrin complexes prepared under alkaline conditions exhibited higher grafting degrees. This difference may be attributed to variations in protein type, polyphenol structure, and reaction conditions, such as pH and temperature. The enhanced grafting observed in URWA–BPs is likely associated with cavitation-induced enhanced mixing and mass transfer, as well as the synergistic effect of sonochemical reactions in the radical system.

### 3.4. Structural Properties of WA–BPs

#### 3.4.1. Surface Hydrophobicity

**Figure 3:**
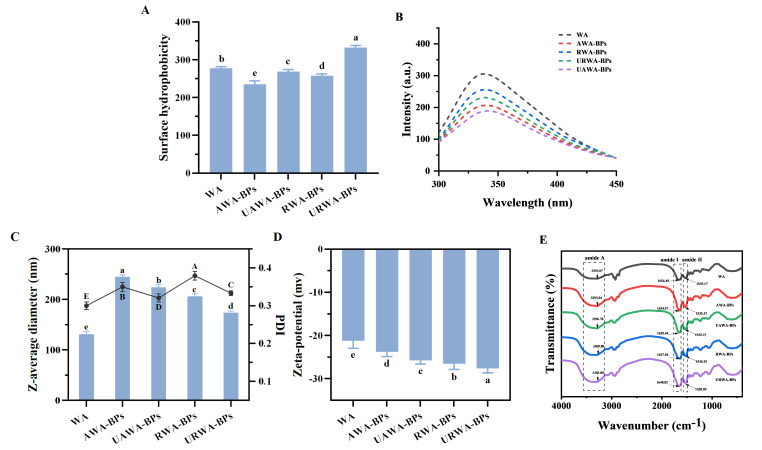
Surface hydrophobicity (H_0_) (**A**), intrinsic fluorescence spectra (**B**), Z-average diameter and PDI (**C**), zeta potential (**D**), and FTIR (**E**) of WA–BPs. Different letters (uppercase and lowercase) indicate significant differences among samples (*p* < 0.05); the same applies hereafter.

The H_0_ of proteins reflects conformational changes in their tertiary structure and is closely related to functional properties such as solubility, foaming, and emulsifying capacity. As shown in Figure 3A, the H_0_ of WA was 277.40 ± 4.36. Compared with WA, AWA–BPs and RWA–BPs exhibited decreased H_0_ values (234.80 ± 9.00 and 257.77 ± 3.51, respectively), suggesting that covalent conjugation under alkaline or radical conditions masked hydrophobic sites. The H_0_ values of UAWA–BPs and URWA–BPs were 268.44 ± 4.58 and 332.13 ± 5.51, respectively, both higher than those of AWA–BPs and RWA–BPs, indicating enhanced exposure of hydrophobic regions under ultrasound, particularly in radical systems. These results indicate that ultrasound influences protein structural rearrangement during conjugation, likely through cavitation-induced enhancement of molecular mobility and partial unfolding. Similar trends have been reported by Yue et al. [44]. Covalent interactions between polyphenols and proteins can further alter the distribution of surface polar and nonpolar groups, thereby affecting H_0_ [45].

#### 3.4.2. Intrinsic Fluorescence Spectroscopy

Intrinsic fluorescence spectroscopy provides insights into protein conformational changes induced by polyphenol binding. The fluorescence signal mainly originates from the Trp, Tyr, and Phe residues [46]. Changes in protein tertiary structure can alter the polarity of the microenvironment around these residues, resulting in decreased fluorescence intensity or a red shift in the emission peak [47]. As shown in Figure 3B, the fluorescence intensity of WA–BPs decreased markedly compared with WA, indicating significant quenching of intrinsic fluorescence after grafting with BPs [48]. Similar results were reported by Guo et al. [49], who observed fluorescence quenching in protein–polyphenol conjugates prepared via radical conjugation methods, indicating that polyphenol binding induces structural rearrangement and reduces intrinsic fluorescence. The fluorescence intensity followed the order: RWA–BPs > URWA–BPs > AWA–BPs > UAWA–BPs. This trend indicates that the extent of fluorescence quenching varied with the modification method, with ultrasound treatments generally enhancing the quenching effect. Combined with the results in Section 3.3, this suggests that more Trp residues participated in covalent binding under ultrasound treatment, resulting in stronger fluorescence quenching. In addition, a slight red shift (3–5 nm) in λmax was observed after modification, suggesting increased polarity of the Trp microenvironment and further confirming conformational changes in the protein.

#### 3.4.3. SEM Analysis

**Figure 4:**
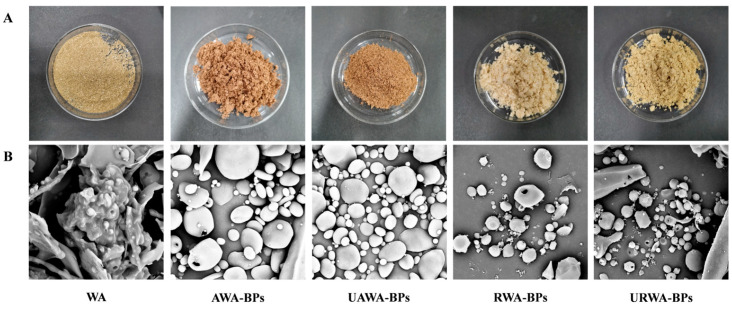
Macroscopic appearance (**A**) and SEM images (**B**) of WA–BPs.

The macroscopic appearances of AWA–BPs, UAWA–BPs, RWA–BPs, and URWA–BPs are shown in Figure 4A. AWA–BPs and UAWA–BPs exhibited a relatively darker coloration, which may be attributed to enhanced oxidation of BPs under alkaline conditions. To further elucidate the structural differences induced by the modification methods, the microstructures of WA–BPs were examined using SEM (Figure 4B). WA exhibited a dense lamellar or block-like structure with a relatively smooth and compact surface, reflecting its natural aggregation and shrinkage during drying. AWA–BPs displayed relatively regular spherical particles with smooth surfaces, which may be associated with protein unfolding and subsequent crosslinking under alkaline conditions [50]. In comparison, UAWA–BPs exhibited smaller and more uniformly distributed spherical structures. This suggests that ultrasound enhanced molecular dispersion and led to more homogeneous aggregation, likely due to cavitation-induced shear forces and improved mass transfer [13]. RWA–BPs showed irregular, rough, and heterogeneous particles, reflecting extensive structural disruption induced by radical reactions. Similar to UAWA–BPs, URWA–BPs exhibited reduced particle size and more uniform microstructures under ultrasound.

#### 3.4.4. Particle Size, PDI and Zeta Potential

The effects of different modification methods on the structural stability of WA–BPs were evaluated by particle size, PDI, and zeta potential [51,52]. As shown in Figure 3C, the mean particle size of WA was 130.97 ± 5.03 nm, while those of AWA–BPs, UAWA–BPs, RWA–BPs, and URWA–BPs increased to 244.83 ± 3.78 nm, 224.83 ± 4.28 nm, 206.27 ± 5.00 nm, and 174.13 ± 2.80 nm, respectively. The increase in particle size after covalent conjugation suggests enhanced intermolecular interactions and aggregation. Among them, AWA–BPs exhibited the largest particle size (*p* < 0.05), indicating stronger crosslinking and aggregation under alkaline conditions [53]. Ultrasound treatment reduced particle size compared with the corresponding non-ultrasound samples. Specifically, UAWA–BPs showed a smaller size than AWA–BPs, suggesting that ultrasound promoted dispersion by disrupting aggregates. All samples showed PDI values below 0.5, indicating relatively uniform size distribution. The highest PDI of RWA–BPs, consistent with SEM results, implied irregular crosslinking and over-aggregation. As shown in Figure 3D, the zeta potential of WA was −21.23 mV, and the absolute values increased after modification. The zeta potential followed the order WA < AWA–BPs < UAWA–BPs < RWA–BPs < URWA–BPs, indicating enhanced exposure of negatively charged groups and improved colloidal stability. Notably, URWA–BPs exhibited the highest absolute zeta potential, suggesting that radical treatment under ultrasound most effectively enhanced surface charge and dispersion stability.

#### 3.4.5. FTIR Analysis

As shown in Figure 3E, WA exhibited characteristic absorption bands at 3293.07 cm^−1^ (amide A, N–H stretching), 1654.49 cm^−1^ (amide I, C=O stretching), and 1535.17 cm^−1^ (amide II, N–H bending and C–N stretching) [54]. Compared with WA, all WA–BP samples exhibited peak shifts, indicating that polyphenol grafting altered the hydrogen-bonding network [55]. In the amide A region, AWA–BPs (3293.04 cm^−1^) showed a slight red shift, whereas UAWA–BPs, RWA–BPs, and URWA–BPs displayed blue shifts (3296.78–3303.08 cm^−1^), suggesting modifications in the N–H hydrogen-bonding environment, particularly under radical and ultrasound conditions. In the amide I region, the peak of RWA–BPs shifted to 1647.94 cm^−1^, suggesting enhanced interactions with C=O and N–H groups under radical conditions, thereby lowering the vibration energy of the C=O bond [56]. URWA–BPs (1648.82 cm^−1^) retained a similar red-shifted feature, indicating that ultrasound mainly intensified radical-driven interactions without altering the reaction method. AWA–BPs (1535.32 cm^−1^) and UAWA–BPs (1535.37 cm^−1^) showed slight blue shifts in the amide II, indicating weak interactions in the alkaline system with limited influence on C–N vibrations [57]. Similar peak shifts at 3370 cm^−1^ (O–H stretching) and 1538 cm^−1^ (NH^3+^ vibration) reported by Li et al. [13] were consistent with these findings. Red shifts in amide I and blue shifts in amide II observed in the catechin–soy protein system further confirmed the cooperative involvement of C=O, C–N, and N–H groups in polyphenol–protein covalent interactions [58].

### 3.5. Functional Properties of WA–BPs

#### 3.5.1. Solubility

**Figure 5:**
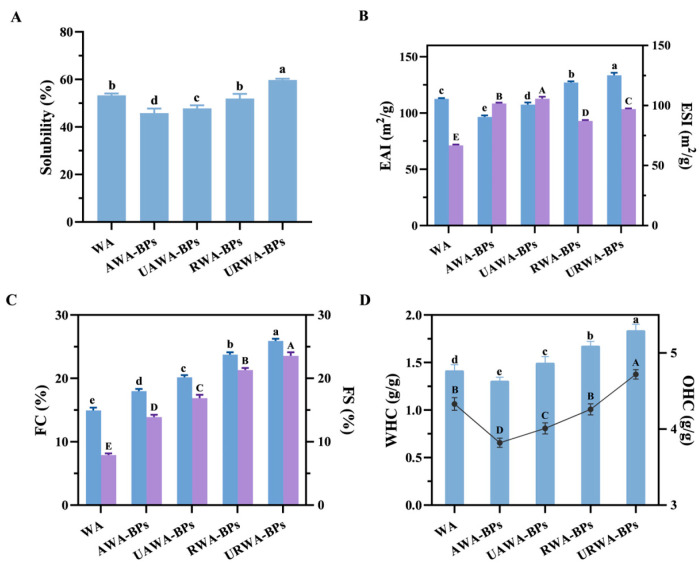
Functional properties of WA–BPs: solubility (**A**), EAI/ESI (**B**), FC/FS (**C**), WHC/OHC (**D**).

The solubility of proteins is closely related to their structural and functional behavior in food systems, playing a crucial role in properties such as emulsification, foaming, and gel formation [59]. Interactions between polyphenols and proteins can markedly influence solubility by altering protein conformation and hydrophilicity. As shown in Figure 5A, the solubility of the samples followed the order URWA–BPs > RWA–BPs > UAWA–BPs > AWA–BPs, indicating that both the grafting method and ultrasonic treatment exerted distinct effects on protein structure. The solubility of WA was 53.33 ± 0.58%, which decreased to 45.83 ± 0.91% for AWA–BPs, likely due to the formation of crosslinked structures that reduced molecular flexibility and water accessibility. RWA–BPs exhibited a solubility of 51.96 ± 0.65%, comparable to that of WA, indicating a balance between structural unfolding and crosslinking effects. In contrast, UAWA–BPs showed a slight increase in solubility (47.83 ± 0.63%) compared with AWA–BPs, while URWA–BPs exhibited the highest solubility (59.74 ± 0.45%), suggesting that ultrasound improved molecular dispersion and protein–water interactions [45].

#### 3.5.2. Emulsifying Properties

Protein–polyphenol complexes can form interfacial films around oil droplets, thereby improving emulsion stability. Their emulsifying properties were evaluated using the EAI and ESI [60]. As shown in Figure 5B, RWA–BPs exhibited a higher EAI (127.08 ± 1.39 m^2^/g) than WA, reflecting enhanced interfacial activity and increased exposure of hydrophobic groups. Cheng et al. [61] similarly reported that conjugates of myofibrillar protein with mulberry polyphenols significantly improved emulsifying activity. Notably, URWA–BPs exhibited the highest EAI (133.16 ± 1.80 m^2^/g), suggesting that the combined ultrasound treatment further enhanced interfacial adsorption capacity. Likewise, UAWA–BPs showed an increased EAI compared with AWA–BPs, indicating improved dispersion and interfacial coverage. In terms of ESI, AWA–BPs and UAWA–BPs exhibited relatively higher stability, whereas RWA–BPs showed a lower ESI, suggesting reduced interfacial film integrity despite enhanced interfacial activity. These results highlight the role of interfacial film structure in emulsion stability. Dong et al. [62] reported that incorporating rutin into soy protein isolate reduced interfacial tension while maintaining interfacial film integrity, thereby enhancing emulsifying activity and stability. Overall, emulsifying behavior is governed by the interplay between molecular dispersion, interfacial adsorption, and structural integrity, consistent with the H_0_ results (Section 3.4.1) [63], with particle size also contributing to emulsion behavior.

#### 3.5.3. Foaming Properties

Many proteins exhibit limited ability to form and stabilize foams, which restricts their functional applications in food processing [64]. Recent studies have shown that the formation of protein–polyphenol complexes can significantly enhance foaming properties [65]. As shown in Figure 5C, both FC and FS increased significantly after polyphenol grafting (*p* < 0.05), following the order URWA–BPs > RWA–BPs > UAWA–BPs > AWA–BPs > WA. Compared with WA (FC: 14.93 ± 0.29%; FS: 7.91 ± 0.11%), all modified samples exhibited enhanced foaming properties, with URWA–BPs showing the highest values (FC: 25.91 ± 0.36%; FS: 23.57 ± 0.53%). This improvement can be attributed to covalent interactions between BPs and WA, which enhance molecular flexibility and interfacial film formation, thereby facilitating protein adsorption and stabilizing the air–water interface [66]. Moreover, ultrasonic cavitation contributes to improved interfacial behavior during foam formation [67].

#### 3.5.4. Water-Holding and Oil-Holding Capacities

WHC and OHC are key functional properties associated with protein–water and protein–oil interactions. As shown in Figure 5D, WHC and OHC exhibited similar trends among all samples. Compared with WA (WHC: 1.42 ± 0.12 g/g; OHC: 4.33 ± 0.30 g/g), AWA–BPs showed reduced capacities, whereas all other modified samples exhibited enhanced values, following the order URWA–BPs > RWA–BPs > UAWA–BPs > WA > AWA–BPs. Notably, URWA–BPs showed the highest WHC (1.84 ± 0.08 g/g) and OHC (4.72 ± 0.22 g/g). The reduced WHC of AWA–BPs may be related to the formation of crosslinked aggregates that limit water-binding sites [19]. In contrast, the enhanced WHC and OHC in other samples suggest improved molecular interactions with water and oil, likely associated with structural modification and increased exposure of functional groups. Moreover, OHC is also closely related to protein structure and the exposure of hydrophobic groups [68].

#### 3.5.5. Antioxidant Capacity

**Figure 6:**
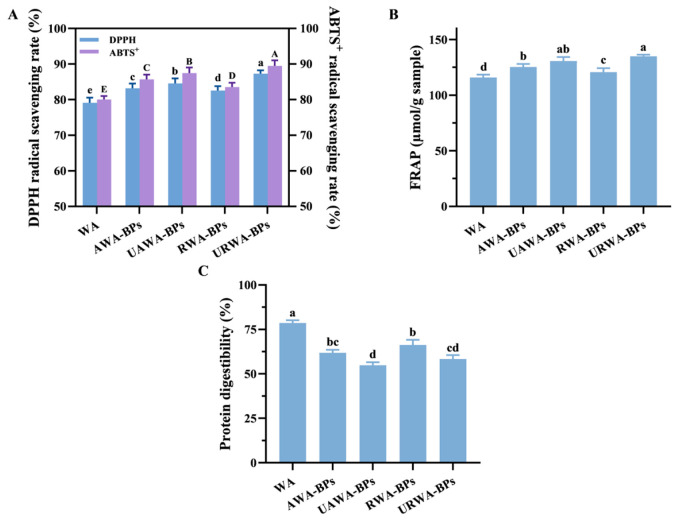
Antioxidant and digestibility properties of WA–BPs: DPPH and ABTS^+^ scavenging activities (**A**), FRAP (**B**), and in vitro protein digestibility (**C**).

Grafting of polyphenols has been widely reported to enhance the antioxidant properties of proteins by improving their radical scavenging capacity [69,70]. In this study, antioxidant capacity was evaluated using DPPH, ABTS^+^, and FRAP assays [71]. As shown in Figure 6A,B, WA exhibited moderate antioxidant activity, with DPPH and ABTS^+^ scavenging rates of 79.16 ± 1.40% and 80.07 ± 0.95%, respectively, and an FRAP value of 115.91 ± 2.50 μmol/g. After BP grafting, all modified samples showed significantly enhanced antioxidant activity (*p* < 0.05), following the order URWA–BPs > UAWA–BPs > AWA–BPs > RWA–BPs > WA. URWA–BPs exhibited the highest antioxidant activity, with DPPH and ABTS^+^ scavenging rates of 87.32 ± 0.97% and 89.46 ± 1.59%, and an FRAP value of 135.00 ± 2.75 μmol/g, corresponding to increases of approximately 10.3%, 11.7%, and 16.5%, respectively, compared with WA. UAWA–BPs also showed notable improvements, whereas RWA–BPs exhibited relatively smaller increases. This enhancement can be attributed to the introduction of phenolic hydroxyl groups through covalent conjugation, which improves electron-donating capacity and radical-scavenging efficiency [72]. The superior performance of URWA–BPs further suggests a synergistic effect between covalent modification and ultrasound-induced structural regulation, facilitating more effective exposure of active sites [73].

### 3.6. In Vitro Protein Digestibility of WA–BPs

Protein digestibility was used to evaluate the nutritional availability and structural stability of protein–polyphenol complexes. As shown in Figure 6C, the digestibility of WA was 78.6 ± 1.5%. After BP grafting, all WA–BP samples showed significantly reduced digestibility (*p* < 0.05), indicating enhanced resistance to gastrointestinal digestion [13]. The digestibility followed the order RWA–BPs > AWA–BPs > URWA–BPs > UAWA–BPs, with values of 66.30 ± 2.80%, 61.90 ± 1.60%, 58.40 ± 2.10%, and 54.90 ± 1.60%, respectively. This reduction in digestibility can be attributed to covalent bonding between BPs and WA, which likely shields protease recognition sites and decreases the accessibility of hydrolysis regions [74]. UAWA–BPs and URWA–BPs showed lower digestibility, which may be associated with ultrasound-modulated covalent assembly, where changes in crosslinking topology and aggregation state result in a denser network with reduced enzyme accessibility [75].

### 3.7. Molecular Docking Analysis

**Figure 7:**
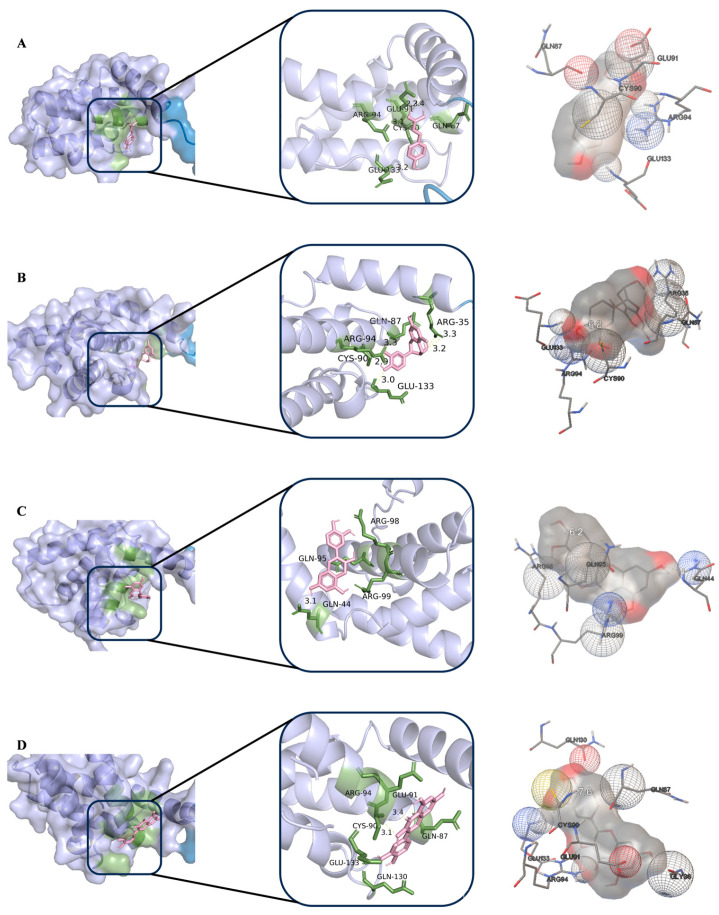
Molecular docking of representative components of BPs with WA. Binding modes of p-coumaric acid (**A**), catechin (**B**), epicatechin (**C**), and quercetin (**D**) in complex with WA. From left to right: overall binding position on the protein surface, enlarged view of the binding pocket (green residues), and 3D interaction diagrams.

Molecular docking results showed that the binding free energies between representative BP components, including p-coumaric acid, catechin, epicatechin, and quercetin, and WA were −5.3, −6.8, −6.2, and −7.6 kcal/mol, respectively, all below −5 kcal/mol, indicating favorable binding affinity (Figure 7). Among them, quercetin exhibited the strongest binding affinity, which may be attributed to its planar conjugated structure and multiple hydroxyl groups that favor stable interactions with the receptor. In contrast, p-coumaric acid showed relatively weaker binding affinity, likely due to its simpler structure and fewer interaction sites [76]. Docking conformations revealed that these phenolic compounds predominantly occupied potential binding pockets on the WA surface and formed relatively stable non-covalent interaction networks with surrounding amino acid residues such as ARG94, GLN87, CYS90, and GLU133 through hydrogen bonding and hydrophobic interactions. Positively charged or polar residues, including ARG94 and GLN87, may stabilize phenolic hydroxyl groups through hydrogen-bond interactions, while the hydrophobic regions surrounding the aromatic rings could further enhance ligand-binding stability. In addition, the sulfhydryl group (–SH) of CYS90 was considered a potential reactive site for quinone-mediated covalent conjugation under oxidative conditions, as thiol groups of cysteine residues are highly susceptible to nucleophilic addition reactions with oxidized quinone intermediates [77]. Such interactions may facilitate the enrichment and spatial proximity of polyphenols on the protein surface, which could promote their oxidation into quinone intermediates under oxidative conditions, thereby enabling subsequent reactions with nucleophilic residues in WA [78]. Furthermore, ultrasound treatment may partially unfold WA, thereby exposing buried hydrophobic regions and reactive amino acid residues, enhancing the accessibility of binding sites and strengthening the interaction between WA and BPs [79]. Collectively, these results support the potential feasibility of WA–BP conjugation from a molecular interaction perspective and provide indirect evidence for the proposed oxidation-mediated covalent binding mechanism.

## 4. Discussion

This study investigated the structural and functional properties of WA following ultrasound-assisted alkaline and radical covalent conjugation with BPs. Compared with single-polyphenol systems and FPs, BPs derived from jujube pomace, as a multicomponent system with higher structural complexity and diverse reactive characteristics, may exhibit more complex interaction patterns and covalent assembly behaviors with WA. The observed changes in surface hydrophobicity, intrinsic fluorescence, and secondary structure suggest that grafting of BPs altered the conformational organization of WA, thereby contributing to the enhancement of its functional performance. Ultrasound treatment exerted distinct regulatory effects on WA–BPs under alkaline and radical conjugation methods. Among all treatments, URWA–BPs exhibited the most pronounced improvement in functional properties, which may be attributed to ultrasonic cavitation-induced protein unfolding, increased exposure of reactive sites, and enhanced oxidation of phenolic hydroxyl groups, thereby promoting covalent conjugation. In contrast, UAWA–BPs showed reduced in vitro digestibility, suggesting that the alkaline conjugation method may favor the formation of a more compact or stable conjugated structure. Such structural stabilization could limit the accessibility of protease-sensitive sites, thereby reducing enzymatic hydrolysis. These findings indicate that the functional and digestive properties of WA–BPs were strongly influenced by the specific covalent conjugation method. Molecular docking revealed favorable interactions between representative components of BPs and WA through hydrogen bonding and hydrophobic interactions involving residues such as ARG94, GLN87, CYS90, and GLU133, suggesting that these interactions may facilitate subsequent oxidation-mediated covalent conjugation. This study provided insights into the differential regulatory effects of ultrasound during protein–polyphenol covalent assembly and supported the development of functional plant protein ingredients. Future studies should further elucidate the molecular mechanisms underlying these effects and evaluate the performance of the conjugates in complex food matrices.

## Data Availability

The original contributions presented in this study are included in the article/Supplementary Material. Further inquiries can be directed to the corresponding authors.

## Supplementary Materials

The following supporting information can be downloaded at: https://www.mdpi.com/article/10.3390/foods15112033/s1, Figure S1: Effects of NaOH concentration (A), S/L ratio (B), temperature (C), and ultrasonic power (D) on the extraction yield of BPs; Figure S2: HPLC chromatograms of mixed polyphenol standards and the extracted sample at 280 nm; Table S1: Gradient elution program for HPLC analysis of BPs; Table S2: Orthogonal test factor levels table; Table S3: Orthogonal test results; Table S4: Composition and content of BPs in jujube pomace; Table S5: Basic composition table of walnut meal; Table S6: Amino acid composition and contents of WA.

## Abbreviations

WA	Walnut albumin
BPs	Bound polyphenols
FPs	Free polyphenols
DW	Dry weight
AWA–BPs	Alkaline-treated WA–BP conjugates
UAWA–BPs	Ultrasound-assisted alkaline WA–BP conjugates
RWA–BPs	Radical-treated WA–BP conjugates
URWA–BPs	Ultrasound-assisted radical WA–BP conjugates
FTIR	Fourier transform infrared spectroscopy
SEM	Scanning electron microscopy
HPLC	High-performance liquid chromatography
H_0_	Surface hydrophobicity
PDI	Polydispersity index
–NH_2_	Free amino groups
SDS	Sodium dodecyl sulfate
EAI	Emulsifying activity index
ESI	Emulsifying stability index
FC	Foaming capacity
FS	Foam stability
DPPH	2,2-diphenyl-1-picrylhydrazyl
ABTS^+^	2,2′-azinobis-(3-ethylbenzothiazoline-6-sulfonic acid) radical cation
FRAP	Ferric reducing antioxidant power
WHC	Water-holding capacity
OHC	Oil-holding capacity
SSF	Simulated salivary fluid
SGF	Simulated gastric fluid
SIF	Simulated intestinal fluid
TCA	Trichloroacetic acid
BCA	Bicinchoninic acid
