# Supplementary material for: Ultrasound-Assisted Covalent Conjugation of Walnut Albumin with Bound Polyphenols: Structural Modulation and Functional Enhancement
Source: Foods. 2026 Jun 5;15(11):2033. doi: 10.3390/foods15112033 (PMC13257139; doi:10.3390/foods15112033)
Supplement: Supplementary file 1 [file foods-15-02033-s001.zip › foods-4362821-supplementary.pdf]

Ultrasound-assisted covalent conjugation of walnut albumin with bound polyphenols: Structural modulation and functional enhancement

1. Single-factor experiments

The BPs yield increased with rising NaOH concentration, reaching a maximum at 1.2 mol/L, and then declined at higher concentrations. Moderate alkalinity promoted the cleavage of linkages between BPs and cell wall components, whereas excessive alkalinity led to structural degradation of polyphenols. As the solid-to-liquid (S/L) ratio increased from 1:10 to 1:20 (g/mL), the release of BPs significantly improved; however, no further increase was observed beyond this point, indicating that 1:20 was the optimal ratio. The BPs yield peaked at 80 °C, while higher temperatures resulted in oxidative losses. Similarly, the extraction rate reached its maximum at an ultrasonic power of 200 W and slightly decreased thereafter. The optimal extraction conditions were determined as follows: NaOH concentration of 1.2 mol/L, S/L ratio of 1:20 (g/mL), extraction temperature of 80 °C, and ultrasonic power of 200 W.

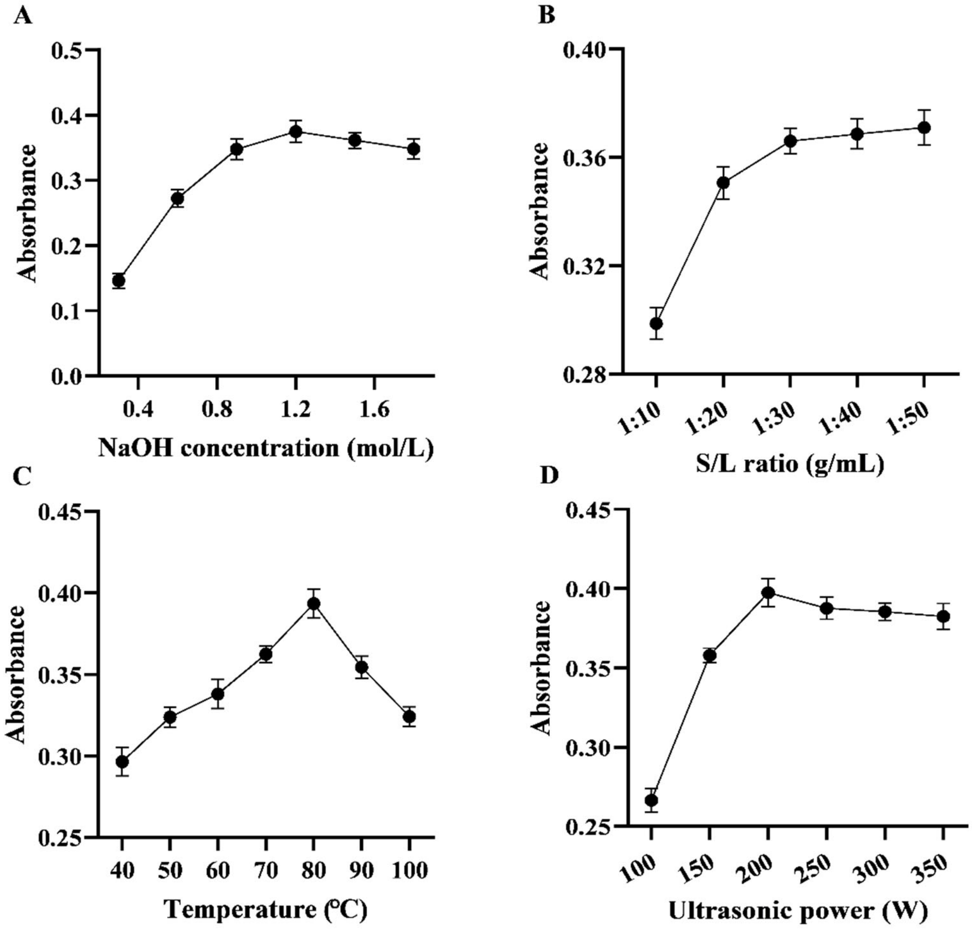

Figure S1. Effects of NaOH concentration (A), S/L ratio (B), temperature (C), and ultrasonic power (D) on the extraction yield of BPs.

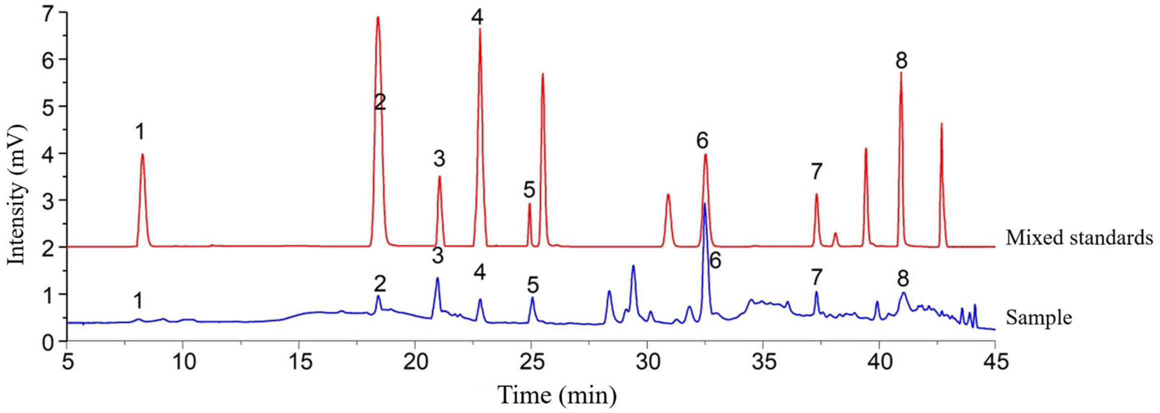

Figure S2. HPLC chromatograms of mixed polyphenol standards and the extracted sample at 280 nm.

Table S1. Gradient elution program for HPLC analysis of BPs.

Mobile phase A: Methanol
Mobile phase B: 0.5% (v/v) formic acid in water
Flow rate: 0.7 mL/min
Column temperature: 30 °C

Time (min)	%B
0.0	90
5.0	85
6.0	80
10.0	80
11.0	75
15.0	60
25.0	60
38.0	41
48.0	37
49.0	34
59.0	33
63.0	0
73.0	0
74.0	90
85.0	90

2. Orthogonal experiments

As shown in Tables S2–S3, the order of factors influencing the extraction efficiency of BPs from jujube pomace was as follows: S/L ratio (B) > extraction temperature (C) > ultrasonic power (D) > NaOH concentration (A). Range analysis indicated that the optimal combination was $A_1B_4C_3D_4$.

Table S2. Orthogonal test factor levels table

Level	Factors			
	**A: NaOH concentration (mol/L)**	**B: S/L ratio (g/mL)**	**C: Temperature (°C)**	**D: Ultrasonic power (W)**
1	1.02	1:14	74	150
2	1.14	1:18	78	200
3	1.36	1:22	82	250
4	1.48	1:26	86	300

Table S3. Orthogonal test results

Run	A	B	C	D	A_{746}
1	1	1	1	1	0.273 ± 0.017
2	1	2	2	2	0.416 ± 0.021
3	1	3	3	3	0.424 ± 0.016
4	1	4	4	4	0.418 ± 0.008
5	2	1	2	3	0.291 ± 0.022
6	2	2	1	4	0.327 ± 0.015
7	2	3	4	1	0.342 ± 0.031
8	2	4	3	2	0.387 ± 0.030
9	3	1	3	4	0.360 ± 0.027
10	3	2	4	3	0.414 ± 0.014
11	3	3	1	2	0.325 ± 0.027
12	3	4	2	1	0.366 ± 0.019
13	4	1	4	2	0.323 ± 0.025
14	4	2	3	1	0.345 ± 0.016
15	4	3	2	4	0.409 ± 0.028
16	4	4	1	3	0.351 ± 0.014
k1	0.383	0.312	0.319	0.332	
k2	0.337	0.376	0.370	0.363	
k3	0.366	0.375	0.379	0.370	
k4	0.357	0.380	0.374	0.379	
R value	0.046	0.068	0.060	0.047	

Table S4. Composition and content of BPs in jujube pomace

Peak No.	Compound	Content (μg/g DW)
1	Gallic acid	0.16 ± 0.04^{g}
2	Protocatechuic acid	1.28 ± 0.13^{d}
3	Catechin	6.31 ± 0.35^{b}
4	p-Hydroxybenzoic acid	0.42 ± 0.05^{f}
5	Epicatechin	3.77 ± 0.52^{c}
6	p-Coumaric acid	19.85 ± 1.21^{a}
7	Rutin	0.94 ± 0.12^{e}
8	Quercetin	1.16 ± 0.03^{d}

Note: Different lowercase letters indicate significant differences among samples ($P < 0.05$).

Table S5. Basic composition table of walnut meal

Sample	Protein (%)	Fat (%)	Ash (%)	Moisture (%)
Walnut meal	47.67 ± 0.06	25.42 ± 0.07	5.26 ± 0.19	8.68 ± 0.17

Table S6. Amino acid composition and contents of WA.

Amino acid	Content (mg/g DW)	Amino acid	Content (mg/g DW)
Aspartic acid (Asp)	0.70±0.11	Methionine (Met)*	0.04±0.01
Threonine (Thr)*	0.26±0.03	Isoleucine (Ile)*	0.15±0.07
Serine (Ser)	0.29±0.05	Leucine (Leu)*	0.29±0.06
Glutamic acid (Glu)	1.67±0.03	Tyrosine (Tyr)	0.20±0.03
Proline (Pro)	0.02±0.01	Phenylalanine (Phe)*	0.20±0.01
Glycine (Gly)	0.32±0.07	Lysine (Lys)*	0.21±0.04
Alanine (Ala)	0.30±0.06	Histidine (His)	0.14±0.02
Cystine (Cys)	0.08±0.01	Arginine (Arg)	0.95±0.06
Valine (Val)*	0.23±0.03	Total amino acids	6.15±0.22

Note: * Essential amino acid.
